# Successful Management of a Huge Pulmonary Hydatid Cyst in a Young Child: A Case Report

**DOI:** 10.34763/jmotherandchild.20263001.d-26-00005

**Published:** 2026-04-30

**Authors:** Manel Kammoun, Saloua Ammar, Hela Fendri, Hayet Zitouni, Riadh Mhiri, Anouar Jarraya

**Affiliations:** Pediatric Anesthesia Department at Hedi Chaker University Hospital, Sfax, Tunisia; Pediatric Surgery Department at Hedi Chaker University Hospital, Sfax, Tunisia; Radiology Department at Hedi Chaker University Hospital, Sfax, Tunisia

**Keywords:** pulmonary hydatid cyst, risk management, paediatric morbidity

## Abstract

Hydatid cyst disease is a parasitic disease induced by Echinococcus granulosus, which is still endemic in northern African countries. We report the case of a late-discovered pulmonary hydatid cyst in a four-year-old boy revealed by recent dyspnoea. The chest X-ray showed a complete white-out of the left hemithorax with mediastinal deviation, and the thoracoabdominal CT scan showed a giant pulmonary hydatid cyst encompassing the entire left lung associated with a hepatic cyst. An emergent superior pulmonary lobe resection was performed under deep general anaesthesia and selective intubation to prevent contralateral lung inundation. The evolution was favourable postoperatively.

## Introduction

Hydatid cyst disease is a parasitic disease induced by Echinococcus granulosus, which is still endemic in northern African countries. In children, the lung is the main location of the disease, and pulmonary hydatid cysts grow faster and are more prone to rupture than liver hydatid cysts [1]. It is most often diagnosed based on respiratory symptoms such as cough, haemoptysis, or chest pain, as well as incidental detection. However, in low- and middle-income countries, where access to healthcare is particularly challenging in rural areas, complicated presentations of the disease can occur, including cyst rupture in the bronchi or pleural cavity, infection, and/or involvement of multiple sites [2]. The presence of a paediatric giant pulmonary hydatid cyst encompassing the whole left lung field, leading to heart compression and mediastinal deviation, is unusual and needs specific perioperative management, particularly when associated with another enormous cyst in the liver.

## Case presentation

### Patient Information

A 4-year-old boy, from a rural area, with no previous comorbidities, was admitted in the paediatric emergency department for respiratory distress. He had experienced a mild form of a common cold with a runny nose two days earlier.

### Clinical Findings

**Figure 1.**
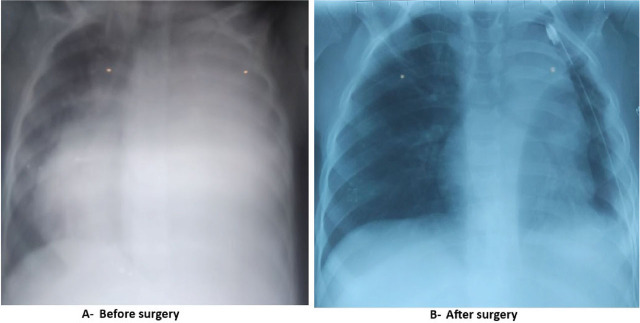
Chest X-Ray.

At admission, the physical examination showed an afebrile child with breathing difficulties and oxygen desaturation at 89%. A hypotension of 85/45 mmHg and tachycardia of 140 beats/min were also observed. Cardiopulmonary auscultation revealed silence on the left lung field with a rightward deviation of heart sounds. The chest X-ray showed a complete white-out of the left hemithorax with severe mediastinal deviation and heart compression (Figure 1). Routine laboratory screening tests and full blood analysis showed anaemia (haemoglobin concentration: 8.8 g/dL; platelet count: 398 × 10^6^/mm^3^) and inflammatory syndrome with hyperleukocytosis and elevated C-reactive protein (WBC: 22.94 × 10^9^; CRP=212.4 mg/L), with no cytolysis, and no ionic or renal disorders.

### Timeline of current episode

The current episode dates back two days, when the boy had a common cold with a runny nose without fever. The child had increasing dyspnoea with breathing struggles, including the rapid onset of nasal flaring, head bobbing, and asymmetric chest in drawing. The child was stabilised by the administration of oxygen at a 4 L/min flow with an infusion of 20 ml/kg normal saline during the stay in the emergency department before he was referred for a thoracoabdominal CT scan.

### Diagnostic assessment

**Figure 2.**
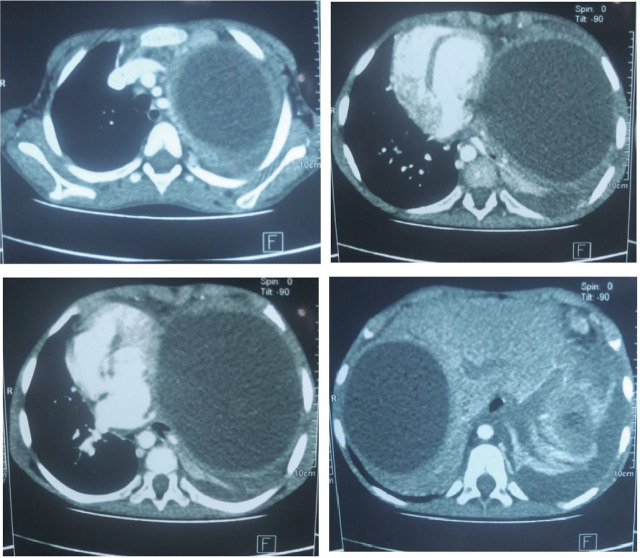
CT scan.

The CT scan showed a large homogeneously hypodense cystic thin-walled lesion occupying the entire left lung field, measuring 114 × 92 × 130 mm. This lesion was responsible for a complete collapse of the left lung, displacing the mediastinum to the right with a Small left pleural effusion with no obvious communication with the cyst. It also showed a large cystic lesion with thin walls in the segments VIII, VII and VI of the liver, measuring 92 × 74 mm, displacing the right portal vein, with no dilation of the bile ducts (Figure 2). Hydatid serological antibodies (IgG) were positive, which confirmed the exposure to Echinococcus granulosus.

### Diagnosis

Given the child's rural background in an endemic zone for Echinococcus granulosus and the highly suggestive appearance on the CT scan and the positive serological tests, the diagnosis of multiple hydatid disease with pulmonary and hepatic locations was made.

### Therapeutic interventions

The patient was referred to the paediatric surgical intensive care unit for quick preoperative optimisation and for management of any probable complications. He received 2 L/min oxygen support via nasal cannula with intravenous preload over three hours before the referral to the operating room for emergent surgery. After correct preoxygenation with spontaneous breathing, the child received 0.3 μg/kg of sufentanil, 5 mg/kg of propofol, and 0.15 mg/kg of cisatracurium. He was gently ventilated via facial mask and intubated with a cuffed N°4 endotracheal tube. The tube was introduced in the main right bronchus and fixed at 18 cm. One-lung ventilation was performed with a tidal volume of 90 ml, a respiratory rate of 30/min, and without PEEP, under strict surveillance of the peak airway pressure (Pmax <25 cm H_2_O). The respiratory rate was adjusted intraoperatively to maintain the end-tidal partial pressure of carbon dioxide (PET CO_2_) in the range of 33–38 mmHg. After lateral thoracotomy, an aspiration, opening of the apex of the cyst and extraction of the proliferative membrane were performed. The whole superior lobe of the lung was destroyed. A lobectomy was decided with placement of a thoracic drainage. At the end of the intervention, the intratracheal tube was withdrawn a few centimetres into the trachea. The two lungs were ventilated, and a PEEP of 5 cm H_2_O was applied to facilitate the lung expansion and to allow the verification of the absence of air leak. The patient received a transfusion of 15 ml/kg over three hours (5 ml/kg/h) and was safely extubated in the operating room before he returned to the ICU.

### Follow-up and outcome of interventions

Postoperatively, the child had stable hemodynamic and respiratory parameters with only 2L/min oxygen via nasal cannula. Multimodal analgesia based on paracetamol, dexamethasone, and continuous infusion of morphine (10mg/24h) was ensured during the first 48 hours following surgery. Incentive spirometry was used to prevent atelectasis and to enhance pulmonary expansion. The patient also received amoxicillin-clavulanic acid and albendazole during the first five days. Feeding was resumed the day after the procedure, and the thoracic drainage was safely removed on the fifth day following the intervention. The evolution was favourable. The patient left the hospital 7 days after the intervention, and he was scheduled for hepatic cyst resection under preventive perioperative coverage using albendazole that will be started 4 days before surgery and will continue for 5 days postoperatively.

### Patient Perspective

During the hospital stay and at discharge, the patient's parents were satisfied with the quality of healthcare.

### Informed Consent and ethics committee approval

Approval from the local ethics committee and informed consent for publication were obtained before the publication of this case report.

## Discussion

The surgical and anaesthetic management of huge pulmonary hydatid cysts remains challenging. In children, huge hydatid cysts are rare and generally explained by delayed or missed diagnosis related to the absence of specific symptoms and the variety of clinical presentations [3]. Furthermore, pulmonary hydatid cysts can grow rapidly, faster than hepatic hydatid cysts, in children because of their more elastic lung tissue, high vascularity, and low negative intrathoracic pressure [4]. On the other hand, they can become rapidly symptomatic due to the compressibility of the lung and the low reserve of oxygen in children, which can lead to rapid oxygen desaturation [4,5].

In our case, the cyst remained asymptomatic despite the compression of the entire pulmonary field and the compression of the contralateral lung by a huge hepatic cyst that pushed the right dome of the diaphragm up. It was discovered after the occurrence of dyspnoea following a minor upper respiratory tract infection, which is not common. Moreover, the rapid evolution can be noticed because the cyst is still homogenously thin-walled and because of the young age of our patient. We should mention that old hydatid cysts are generally pseudo-tumoral or calcified [5]. We also think that the difficult access to healthcare in rural areas of low-resource countries can delay the diagnosis and lead to the late discovery of giant and multiple cysts. Multi-organ involvement, including the lung and liver or other organs, like in our case, can be observed in 6 to 34% [6]. The diagnosis of the disease is made based on radiological examinations such as chest radiography and ultrasound in endemic areas. Computed tomography (CT) scans of the chest and upper abdomen is usually reserved for giant, complicated, multiple cysts, or in cases of diagnostic doubt with other cystic pulmonary lesions [7]. The serological tests may confirm the diagnosis of echinococcosis, but a negative test cannot exclude the disease.

Despite the lack of standardised guidelines, huge pulmonary cysts generally require emergent open surgical intervention, as soon as the diagnosis is made, allowing the removal of the parasite before complications occur. Some precautions should be taken to avoid its spread during surgery. The lung-conservation surgery should always be preferred. Anatomical resections should be considered as a last surgical resort and indicated in case of destroyed or non-functional parenchyma [8,9]. Minimally invasive approaches can be used for peripheral or small lesions with caution to avoid intraoperative dissemination. For our case, percutaneous aspiration, injection, and re-aspiration (PAIR) under computed tomography (CT scan) guidance were discussed with the radiologists, but they were discouraged because of the lack of experience in such rare cases with high risk of spillage, which can lead to an anaphylactic shock, as well as the presence of pulmonary parenchyma destruction that may require lobectomy.

The particularity of this case is that, although the low age of the occurrence of this parasitosis in this child, the volume of the cyst reached a huge size rapidly and led to the destruction of the entire superior lobe of the left lung, requiring an invasive surgical approach and rigorous perioperative follow-up. To prevent the intraoperative dissemination of the disease, a selective intubation and one-lung ventilation should be performed during the removal of the cyst. The main intraoperative risk is the rupture of the cyst due to the intrathoracic hyperpressure caused by mechanical ventilation. This complication can lead to the inundation of the contralateral safe lung that may immediately threaten the child's life [10]. Furthermore, the monitoring of the airway pressures under one-lung ventilation is mandatory to prevent barotrauma in the safe lung and to avoid any intra-abdominal hyperpressure that can lead to the rupture of the associated hepatic cyst. We should also mention that anaphylactic shock can occur during the surgical manipulation of the cyst as well as the perioperative period, even with non-ruptured cysts [11]. Postoperatively, incentive spirometry was efficient in preventing atelectasis as a part of the enhanced recovery program. Other techniques of noninvasive ventilation, such as the Boussignac facial mask or high-flow nasal cannula, should be avoided, as they can result in the recanalization of fistulas, which may prolong the duration of thorax drainage. In huge hydatid cysts, preventive medical treatment, such as Albendazole or praziquantel, is widely recommended after surgery to reduce the chances of recurrence and dissemination of the disease [12]. However, the optimum duration of this therapy is unknown.

### Key messages

- Pulmonary hydatid cysts can grow rapidly in children, and non-specific clinical presentations may delay the diagnosis.
- Late diagnosis of pulmonary echinococcus can lead to lung destruction requiring pulmonary resection.
- Selective intubation is mandatory to prevent the risk of contralateral pulmonary inundation.
- Albendazole is required to prevent the dissemination and/or recurrence of the disease.
